# Real‐world treatment patterns of metastatic non‐small cell lung cancer patients receiving epidermal growth factor receptor tyrosine kinase inhibitors

**DOI:** 10.1002/cam4.4918

**Published:** 2022-06-15

**Authors:** Rahul Shenolikar, Sizhu Liu, Anne Shah, Jenny Tse, Yao Cao, Aimee Near

**Affiliations:** ^1^ AstraZeneca Gaithersburg Maryland USA; ^2^ IQVIA Durham North Carolina USA

**Keywords:** metastasis, non‐small cell lung cancer, target therapy, tyrosine kinase inhibitors

## Abstract

**Background:**

Several epidermal growth factor receptor tyrosine kinase inhibitors (EGFR TKI) have been approved for first‐line (1L) treatment of EGFR‐mutated metastatic non‐small cell lung cancer (mNSCLC) in the United States (US). Real‐world analyses of 1L treatment patterns with EGFR TKIs, including the third‐generation EGFR TKI osimertinib which was most recently approved in 2018, are still sparse.

**Methods:**

This retrospective observational study used data from IQVIA's prescription claims (LRx) and medical claims (Dx) databases. mNSCLC patients newly treated with any EGFR TKI in the 1L setting were identified from January 1, 2015 to April 30, 2020; the first date of EGFR TKI (third‐generation osimertinib, first‐generation [erlotinib, gefitinib], or second‐generation [afatinib, dacomitinib]) was the index date. Treatment patterns were reported in the cohorts stratified by 1L EGFR TKI.

**Results:**

A total of 2505 patients were included in the study (982 osimertinib, 1060 first‐generation, and 463 second‐generation EGFR TKI). Beginning in 2018, osimertinib became the most common 1L EGFR TKI (66.7%) and in early 2020, it accounted for 90.6% of 1L EGFR TKIs. Nearly all patients (>97%) were treated with 1L EGFR TKI monotherapy. Patients with 1L osimertinib had longer treatment duration compared to patients with 1L first‐ or second‐generation EGFR TKI (median months: 17.8 vs. 8.7 vs. 10.5, respectively; log‐rank test for comparisons with osimertinib *p* < 0.0001) over median follow‐up times of 9.8, 20.5, and 19.3 months. 32.5% and 36.3% of the first‐ and second‐generation EGFR TKI cohorts, respectively, had evidence of 2L treatment. Osimertinib monotherapy accounted for the majority of 2L treatments (58.3%/60.7%) and 11.3%/8.9% had 2L chemotherapy or immuno‐oncology therapy following 1L first‐ or second‐generation EGFR TKI.

**Conclusion:**

In this real‐world study of a US claims database, 1L treatment duration was longer with osimertinib compared with other EGFR TKIs. Future studies with longer follow‐up are recommended to understand treatment patterns after progression on EGFR TKIs.

## Graphical abstract

Several epidermal growth factor receptor tyrosine kinase inhibitors (EGFR TKI) have been approved for first‐line (1L) treatment of EGFR‐mutated metastatic non‐small cell lung cancer (mNSCLC) in the United States. This study leveraged prescription and medical claims databases to describe real‐world 1L treatment patterns and found that 1L treatment duration was longer with osimertinib compared with other EGFR TKIs.

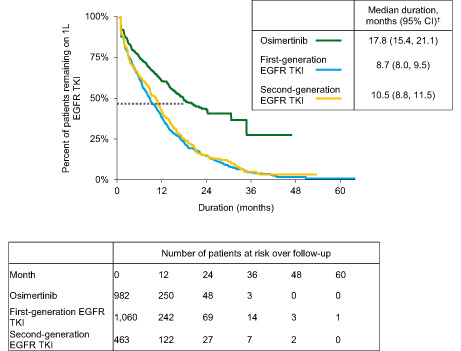

## 1 INTRODUCTION

Prognosis for metastatic non‐small cell lung cancer (mNSCLC) has improved in the past two decades with the identification of certain biomarkers or genetic drivers, allowing for a more tailored therapeutic approach. Current National Comprehensive Cancer Network® (NCCN®) guidelines recommend molecular testing for eligible patients with mNSCLC to allow targeted treatment for actionable mutations, such as mutations in epidermal growth factor receptor (EGFR), to improve outcomes.^1^ The first‐generation epidermal growth factor receptor tyrosine kinase inhibitors (EGFR TKIs) erlotinib and gefitinib were approved by the United States (US) Food and Drug Administration (FDA) for first‐line (1L) treatment of EGFRm patients in May 2013 and July 2015, respectively, after clinical studies showed an improvement in progression‐free survival (PFS) and overall response rate over standard‐of‐care chemotherapy.^2^, ^3^ The second‐generation EGFR TKIs afatinib and dacomitinib received FDA approval in January 2018^4^ and September 2018,^5^ respectively, for 1L treatment of EGFRm patients, after demonstrating either similar or improved survival outcomes compared to gefitinib and erlotinib.^6^

Most patients treated with first‐ or second‐generation EGFR TKIs in 1L generally acquire resistance mutations after a median treatment period of 9–13 months, leading to disease progression.^7^, ^8^ Osimertinib is a third‐generation EGFR TKI that was initially approved in November 2015 in the US for the treatment of patients with T790M‐positive NSCLC, following disease progression on 1L EGFR TKIs. This indication was expanded in April 2018 in the US to 1L treatment of EGFRm patients, based on the FLAURA trial that demonstrated improved PFS with osimertinib compared to first‐generation TKIs, as well as superior overall survival (median, 38.6 months vs. 31.8 months).^9^, ^10^ Consequently, osimertinib is the preferred 1L EGFR TKI as per the NCCN Clinical Practice Guidelines in Oncology (NCCN Guidelines®) in EGFRm mNSCLC.^1^

There is published real world data on first‐ and second‐generation EGFR TKIs^11^, ^12^, ^13^; however, there is no published data on the real‐world use of 1L osimertinib in comparison to other EGFR TKIs. Hence, this retrospective database analysis of mNSCLC patients was conducted to understand treatment patterns in patients treated with EGFR TKIs in the 1L setting, including type and duration of 1L treatment and second‐line (2L) treatments.

## 2 METHODS

### 2.1 Study design

This retrospective observational study used data from IQVIA's prescription claims (LRx) and medical claims (Dx) databases during the study period from January 1, 2014 to May 31, 2020. Patients with mNSCLC who were newly treated with any EGFR TKI in the 1L setting were selected, where initiating an EGFR TKI within 90 days after the earliest observed metastasis diagnosis was used as a proxy for 1L treatment.^14^ The baseline period was 12 months prior to the index date for describing patient demographics and clinical characteristics. A variable follow‐up period (no minimum follow‐up) was used to assess the 1L and 2L treatment duration using all available data.

### 2.2 Study databases

The LRx database captures information on dispensed prescriptions with 92% coverage of prescriptions from the retail channel, 72% coverage of standard mail service, and 76% coverage of long‐term care facilities in the US. The Dx database captures over 1 billion pre‐adjudicated claims and 3 billion records obtained annually from approximately 800,000 office‐based physicians and specialists, with 75% of American Medical Association providers being captured. Medical claims from ambulatory and general health care sites (as well as outpatient clinics associated with hospitals such as rehabilitation, same day surgery, and chemotherapy centers) are also included in the Dx database. All data are Health Insurance Portability and Accountability Act (HIPAA)‐compliant to protect patient privacy. The study dataset was created based on HIPAA‐compliant linking processes using IQVIA's patented and proprietary encryption algorithm.^15^, ^16^, ^17^

### 2.3 Patient selection

**FIGURE 1:**
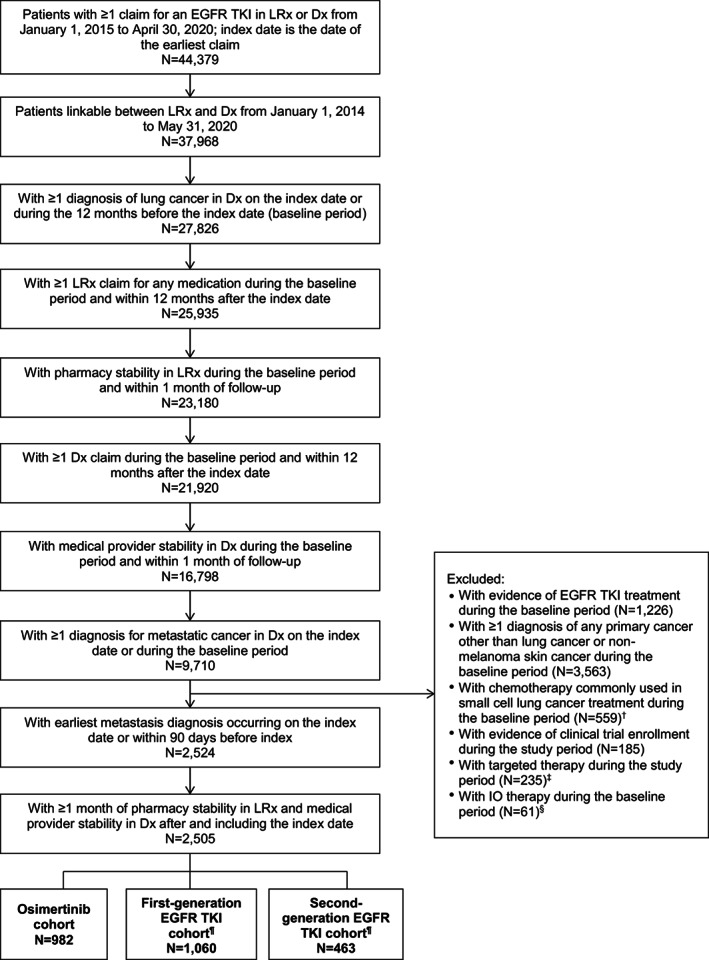
Patient selection. Dx, medical claims database; EGFR TKI, epidermal growth factor receptor tyrosine kinase inhibitor; IO, immuno‐oncology; LRx, prescription claims database. ^†^Chemotherapies commonly used in small cell lung cancer treatment include cisplatin, carboplatin, cyclophosphamide, doxorubicin, etoposide, irinotecan, topotecan, and vincristine. ^‡^Targeted therapies include alectinib, bevacizumab, brigatinib, ceritinib, crizotinib, dabrafenib, entrectinib, larotrectinib, lorlatinib, ramucirumab, and trametinib. ^§^IO therapies include atezolizumab, durvalumab, necitumumab, nivolumab, and pembrolizumab. ^¶^First‐generation EGFR TKIs include erlotinib and gefitinib. Second‐generation EGFR TKIs include afatinib and dacomitinib.

Inclusion and exclusion criteria for patient selection are described in Figure 1. Patients with ≥1 claim for an EGFR TKI (first‐generation: Gefitinib, erlotinib; second‐generation: Afatinib, dacomitinib; third‐generation: Osimertinib) from January 1, 2015 to April 30, 2020 were included. Although osimertinib was only approved for the 1L treatment of EGFRm mNSCLC in 2018, a selection window starting in 2015 was used to capture additional patients with 1L use of first‐ and second‐generation EGFR TKIs. The earliest prescription claim in LRx or drug administration claim in Dx was considered as the index date. All patients treated with an EGFR TKI were assumed to have an EGFR mutation. Patients with ≥1 diagnosis code for lung cancer (International Classification of diseases; ICD‐9: 162.2x–162.9x and ICD‐10: C34) on index date or during the baseline period and earliest metastatic cancer diagnosis (ICD‐9: 196.xx–198.xx and ICD‐10: C77.x–C79.x) on or 90 days before the index date were included.^14^ Other selection criteria are detailed in Figure 1. Patients were stratified into three mutually exclusive cohorts based on their index EGFR TKI (first‐generation EGFR TKI, second‐generation EGFR TKI, or third‐generation EGFR TKI [osimertinib]).

### 2.4 Treatment variables

#### 2.4.1 Treatment regimen

A treatment regimen was defined as a continuous period of medication use during follow‐up. If only one medication was used without any other agents (e.g., chemotherapy or immuno‐oncology [IO] therapy) observed within a 30‐day window including the initial medication dispensing, then it was considered a monotherapy regimen.^14^ If any other agent was initiated within the 30‐day window, the regimen was considered a combination regimen.

#### 2.4.2 Treatment duration

The duration of a treatment regimen was defined as the time from the regimen initiation to the regimen end. The date of regimen initiation was the date of the earliest claim of the medication for a monotherapy regimen and, for combination regimens, the date of regimen initiation was the date of the claim that occurred earliest within the combination. For 1L treatment, the regimen initiation date was the index date. The date of regimen end for monotherapy or combination regimen was the date of regimen discontinuation or the end of follow‐up, whichever occurred earlier. The date of regimen discontinuation was the date of the last claim in the regimen plus days' supply for that claim. Therefore, patients were right censored if they had no evidence of treatment discontinuation by the end of follow‐up.

#### 2.4.3 Treatment discontinuation

Treatment with an EGFR TKI was considered as discontinued when there was a gap of >60 days in its medication supply (i.e., gap after the end of the days' supply of the last fill) during the follow‐up period. The >60‐day gap was chosen for the primary analysis, since this threshold was used in previous studies of NSCLC treatment patterns^18^ and the EGFR TKIs typically have a 30‐day medication supply. A sensitivity analysis using a gap of >90 days in medication supply was also performed.

### 2.5 Study measures and endpoints

Baseline measures of patients included demographic information and clinical characteristics such as categories of the Dartmouth‐Manitoba's Charlson Comorbidity Index (CCI)^19^ (modified to exclude solid tumor). Number of unique metastasis sites were analyzed based on all diagnosis codes of metastasis, observed on the index date or during the baseline period (within 90 days before the index date). Evidence of incident metastasis to the brain or spinal cord was defined as a diagnosis code for metastasis to the brain or spinal cord in the follow‐up period, with no evidence of prior diagnosis on index or during baseline.

The primary endpoints included type of 1L EGFR TKI therapy, discontinuation of treatment, and duration of 1L treatment. Secondary endpoints included 2L treatment patterns: frequency of 2L treatment and type of 2L treatment. Secondary endpoints were only reported for patients who received first‐generation or second‐generation EGFR TKI in 1L, due to limited follow‐up time for the 1L osimertinib cohort.

### 2.6 Statistical analysis

Kaplan–Meier (KM) analysis was used to estimate median and 95% confidence interval for 1L treatment duration; patients without evidence of 1L treatment discontinuation were censored at the end of follow‐up or the end of the study period, whichever occurred earliest. To assess for differences between the KM curves for osimertinib versus first‐generation EGFR TKI, and osimertinib versus second‐generation EGFR TKI, log‐rank tests were used, with *p* < 0.05 considered to be statistically significant.

Follow‐up time was calculated using two approaches: the time from the index date to the end of follow‐up and the reverse‐KM method to estimate the corresponding follow‐up time for the KM analyses of 1L treatment duration. In the reverse‐KM analysis, the event of interest was the end of follow‐up or the end of the study period, whichever occurred first, and patients were censored at 1L treatment discontinuation (defined using a gap of >60 days in medication supply). The reverse‐KM approach describes both the extent and timing of loss to follow‐up and therefore is helpful for interpreting data from an immature survivor function, such as in interim analyses of prospective clinical trials or for evaluating whether there is sufficient follow‐up data in a retrospective database.^20^, ^21^ Given that osimertinib was approved in April 2018 and the study selection window began in 2015, patients in the osimertinib cohort had less available follow‐up time to discontinue 1L treatment and were more likely to have been censored compared to patients in the other EGFR TKI cohorts (i.e., the data for the osimertinib cohort was less complete). To further investigate the impact of the incomplete follow‐up data for the osimertinib cohort, sensitivity analyses of 1L treatment duration were conducted among subgroups of patients defined by minimum treatment duration (≥1 refill of index EGFR TKI), index date (indexed April 2018 or later), or minimum follow‐up duration (≥12 or ≥24 months).

All statistical analyses were performed using SAS version 9.4 (SAS Institute Inc.).

## 3 RESULTS

The study included a total of 2505 mNSCLC patients who received 1L EGFR TKI therapy (982 in osimertinib cohort, 1060 in first‐generation EGFR TKI cohort, and 463 in second‐generation EGFR TKI cohort).

### 3.1 Demographic and clinical characteristics

The demographic and baseline clinical characteristics of the three cohorts are shown in Table 1. Briefly, the median ages of the cohorts ranged from 66 to 69 years, with a higher proportion of patients aged 45–64 years in the osimertinib cohort relative to the first‐ and second‐generation EGFR TKI cohorts (40.0% vs. 31.8% vs. 38.0%, respectively). In all cohorts, about two‐thirds of patients were females, patients were primarily located in the South (29.2%–41.0%), and most patients were commercially‐insured (50.8%–62.9%) or had Medicare (35.3%–45.9%). Patients with 1L osimertinib or other EGFR TKIs had median baseline CCI scores of 1 and 2, respectively. Baseline metastases to bone or bone marrow were most common (47.9%–48.3%), followed by central nervous system (CNS) metastases (32.4%–38.9%). The third most common metastasis site was the lymph nodes for patients with 1L osimertinib (24.2%) and the respiratory system (including metastasis to lung, pleura, mediastinum, and other respiratory sites) for patients with other 1L EGFR TKIs (29.6%–30.7%). During baseline, 38.7% of osimertinib patients had baseline brain or spinal cord metastases compared to 32.2%–34.8% of patients with other EGFR TKIs. The frequencies of incident brain or spinal cord metastases among the patients without these metastases at baseline were 12.8% for the osimertinib cohort, 16.6% for first‐generation EGFR TKI cohort, and 24.5% for the second‐generation EGFR TKI cohort.

**TABLE 1:** Patient demographic and clinical characteristics by index 1L EGFR TKI

Measures	Osimertinib *N* = 982	First‐generation EGFR TKI *N* = 1060	Second‐generation EGFR TKI *N* = 463
Age, years			
Median (Q1, Q3)	66 (59, 75)	69 (61, 78)	68 (59, 75)
Age group, years, *n* (%)			
18–44	33 (3.4)	27 (2.5)	15 (3.2)
45–64	393 (40.0)	337 (31.8)	176 (38.0)
65–74	308 (31.4)	336 (31.7)	150 (32.4)
≥75	248 (25.3)	360 (34.0)	122 (26.3)
Female			
*n* (%)	659 (67.1)	685 (64.6)	302 (65.2)
Geographic region, *n* (%)			
Northeast	192 (19.6)	171 (16.1)	67 (14.5)
Midwest	185 (18.8)	223 (21.0)	88 (19.0)
South	342 (34.8)	309 (29.2)	190 (41.0)
West	241 (24.5)	256 (24.2)	90 (19.4)
Other/unknown	22 (2.2)	101 (9.5)	28 (6.0)
Index year, *n* (%)			
2015	2 (0.2)	335 (31.6)	77 (16.6)
2016	16 (1.6)	340 (32.1)	126 (27.2)
2017	43 (4.4)	266 (25.1)	130 (28.1)
2018	335 (34.1)	93 (8.8)	74 (16.0)
2019	431 (43.9)	22 (2.1)	44 (9.5)
2020	155 (15.8)	4 (0.4)	12 (2.6)
Payer type^a^, *n* (%)			
Commercial	610 (62.1)	538 (50.8)	291 (62.9)
Medicare	347 (35.3)	487 (45.9)	169 (36.5)
Other	25 (2.5)	35 (3.3)	3 (0.6)
Modified CCI^b^			
Median (Q1, Q3)	1 (0, 3)	2 (1, 3)	2 (1, 3)
Number of unique metastasis sites			
Median (Q1, Q3)	1 (1, 2)	1 (1, 2)	1 (1, 2)
Location of metastases^c^, *n* (%)			
Bone or bone marrow	474 (48.3)	474 (44.7)	222 (47.9)
CNS	382 (38.9)	343 (32.4)	165 (35.6)
Brain or spinal cord	380 (38.7)	341 (32.2)	161 (34.8)
Other CNS	9 (0.9)	12 (1.1)	8 (1.7)
Lymph nodes	238 (24.2)	201 (19.0)	89 (19.2)
Respiratory system	234 (23.8)	314 (29.6)	142 (30.7)
Visceral	125 (12.7)	112 (10.6)	56 (12.1)
Breast/reproductive organs/skin	4 (0.4)	5 (0.5)	2 (0.4)
Other unspecified sites	94 (9.6)	95 (9.0)	40 (8.6)
Patients with incident brain or spinal cord metastasis during follow‐up^d^			
*N* (%)	77 (12.8)	119 (16.2)	74 (24.5)

Abbreviations: CCI, Charlson Comorbidity Index; CNS, central nervous system; EGFR TKI, epidermal growth factor receptor tyrosine kinase inhibitor; Q1, quartile 1; Q3, quartile 3.

^a^ Medicare includes Medicare and Medicare Part D. Other payer type includes Medicaid, cash payments, and unspecified.

^b^ Modified to exclude any malignancy and metastatic solid tumor.

^c^ Based on secondary cancer diagnosis codes on the index date or within the 90 days before the index date.

^d^ Incident metastasis to brain or spinal cord during the follow‐up period is defined as a diagnosis code for metastasis to brain or spinal cord in the follow‐up period, with no evidence of prior diagnosis during baseline. Percentages are calculated using the number of patients without baseline brain or spinal cord metastasis as the denominator.

### 3.2 1L treatment patterns

**FIGURE 2:**
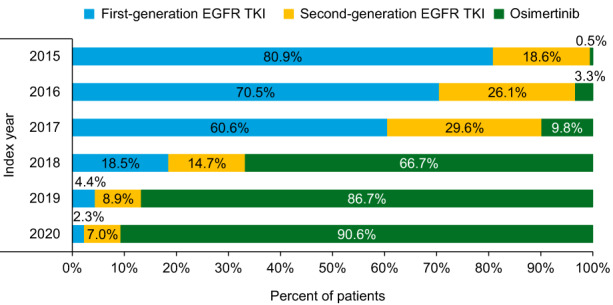
Distribution of 1L EGFR TKIs by index year for each cohort from 2015–2020. EGFR TKI, epidermal growth factor receptor tyrosine kinase inhibitor.

First‐generation EGFR TKIs accounted for most 1L treatments from 2015 to 2017 (60.6%–80.9%) and second‐generation EGFR TKIs accounted for 18.6%–29.6% of 1L EGFR TKIs. Osimertinib was the most common 1L EGFR TKI from 2018 and onwards, consistent with its FDA approval in 2018. In 2020 (January through April), osimertinib accounted for 90.6% of all 1L EGFR TKIs (Figure 2). Across the three cohorts, nearly all (>97%) 1L EGFR TKI treatments were used as monotherapies (Table 2). Among the few patients (*N* = 26, 1.0%) with evidence of EGFR TKI combination therapy, there were 21 (80.8%) patients with EGFR TKI plus EGFR TKI, 3 (11.5%) patients with an EGFR TKI plus chemotherapy, and 2 (7.7%) patients with EGFR TKI plus IO therapy.

**TABLE 2:** Types of 1L EGFR TKI treatments by index 1L EGFR TKI

Type of treatment	Osimertinib *N* = 982	First‐generation EGFR TKI *N* = 1060	Second‐generation EGFR TKI *N* = 463
EGFR TKI monotherapy			
*N* (%)	979 (99.7)	1048 (98.9)	452 (97.6)
Osimertinib	979 (99.7)	0	0
Gefitinib	0	65 (6.1)	0
Erlotinib	0	983 (92.7)	0
Afatinib	0	0	450 (97.2)
Dacomitinib	0	0	2 (0.4)
EGFR TKI combination therapy			
*N* (%)	3 (0.3)	12 (1.1)	11 (2.4)
EGFR TKI + EGFR TKI	3 (0.3)	9 (0.8)	9 (1.9)
EGFR TKI + chemotherapy	0	1 (0.1)	2 (0.4)
EGFR TKI + IO therapy	0	2 (0.2)	0

Abbreviations: EGFR TKI, epidermal growth factor receptor tyrosine kinase inhibitor; IO, immuno‐oncology.

**FIGURE 3:**
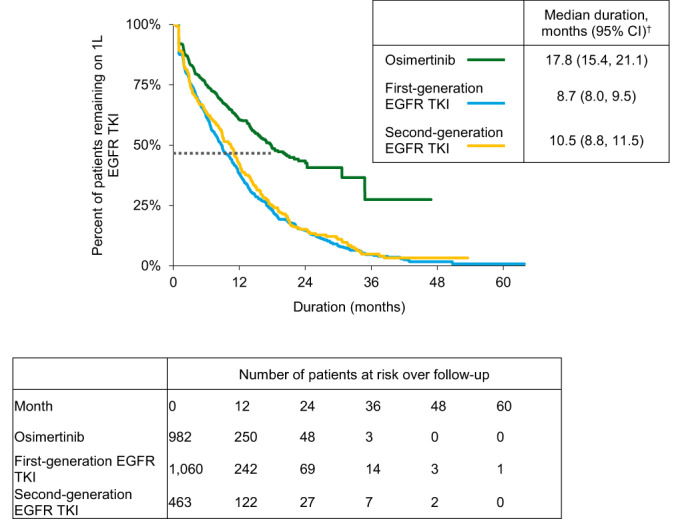
Kaplan–Meier analyses for 1L EGFR TKI treatment duration. 1L, first line therapy; CI, confidence interval; EGFR TKI, epidermal growth factor receptor tyrosine kinase inhibitor. ^†^All log‐rank *p*‐values for comparisons with osimertinib are <0.0001.

Based on reverse‐KM analysis, the osimertinib cohort had the shortest median follow‐up time (9.8 months) compared to patients with first‐generation (20.5 months) or second‐generation (19.3 months) EGFR TKI. During follow‐up, the median 1L treatment duration was significantly longer for osimertinib (17.8 months), compared with second‐generation EGFR TKI (10.5 months) and first‐generation EGFR TKI (8.7 months; log‐rank *p* < 0.0001 for comparisons of first‐ and second‐generation EGFR TKIs with osimertinib) (Figure 3). The proportion of patients with 1L treatment discontinuation by 12 months was 29.5%, 49.6%, and 46.7% for the osimertinib, first‐generation EGFR TKI, and second‐generation EGFR TKI cohorts, respectively (Table S1).

In the sensitivity analysis using a >90‐day gap to define 1L EGFR TKI discontinuation, duration of osimertinib treatment was still longer and fewer patients discontinued 1L treatment. Using the >90‐day gap definition, the median durations of 1L osimertinib, second‐generation EGFR TKI, and first‐generation EGFR TKI were 24.3, 11.7, and 10.7 months, respectively. By 12 months, 23.3%, 42.7%, and 39.5% of patients in the osimertinib, first‐generation, and second‐generation EGFR TKI cohorts had discontinued 1L treatment, respectively (Table S1). In additional sensitivity analyses to address the shorter follow‐up time of the osimertinib cohort (i.e., ≥1 refill of index EGFR TKI, indexed April 2018 or later [to ensure all EGFR TKI cohorts have similar duration of follow up], or minimum follow‐up duration ≥12 or ≥24 months), the duration of 1L osimertinib was consistently longer than the duration of the other EGFR TKIs (Table S1).

### 3.3 2L treatment patterns

During the available follow‐up time, 32.5% of the first‐generation EGFR TKI cohort and 36.3% of the second‐generation EGFR TKI cohort used 2L treatment (Table 3). Among patients with 2L treatment, 1L EGFR TKI was typically followed by 2L EGFR TKI monotherapy (88.4%–89.3%) in both cohorts. After first‐ or second‐generation EGFR TKI in 1L, osimertinib monotherapy accounted for the majority of 2L treatments (58.3%–60.7%). Non‐EGFR TKI treatments such as chemotherapy or IO therapy were less common (8.9%–11.3%) (Table 3).

**TABLE 3:** Types of 2L treatments among patients with 1L first‐ or second‐generation EGFR TKI^a^

Type of treatment	First‐generation EGFR TKI *N* = 1060	Second‐generation EGFR TKI *N* = 463
Patients with 2L treatment		
*N*, (%)	345 (32.5)	168 (36.3)
Type of second‐line treatment, among patients with second‐line treatment (*n*, %)		
EGFR TKI monotherapy	305 (88.4)	150 (89.3)
Same as 1L EGFR TKI	50 (14.5)	19 (11.3)
Osimertinib	201 (58.3)	102 (60.7)
Other EGFR TKI	104 (30.1)	48 (28.6)
Gefitinib	13 (3.8)	1 (0.6)
Erlotinib	50 (14.5)	26 (15.5)
Afatinib	41 (11.9)	20 (11.9)
Dacomitinib	0	1 (0.6)
Non‐EGFR TKI therapy only	39 (11.3)	15 (8.9)
Chemotherapy	22 (6.4)	7 (4.2)
IO therapy	13 (3.8)	5 (3.0)
Chemotherapy + chemotherapy	2 (0.6)	0
IO therapy + chemotherapy	2 (0.6)	3 (1.8)
EGFR TKI combination therapy	1 (0.3)	3 (1.8)
EGFR TKI + EGFR TKI	1 (0.3)	1 (0.6)
EGFR TKI + chemotherapy	0	0
EGFR TKI + IO therapy	0	2 (1.2)

Abbreviations: 1L, first‐line; 2L, second‐line; EGFR TKI, epidermal growth factor receptor tyrosine kinase inhibitor; IO, immuno‐oncology.

^a^ 2L treatment patterns were not reported for the osimertinib cohort due patients' limited follow‐up time.

## 4 DISCUSSION

This retrospective study using real‐world US claims data investigated a total of 2505 mNSCLC patients newly initiating 1L EGFR TKIs and described key patient characteristics and treatment patterns. Median ages of patients were similar across all three cohorts and the patients were predominantly female, which is consistent with the populations reported in other real‐world studies for 1L EGFR TKI use^11^, ^12^, ^13^ and with the prevalence of EGFR mutations in women.^22^

This study has shown patterns of EGFR TKI use changed over time with a shift from the prescription of first‐ and second‐generation EGFR TKIs to third‐generation EGFR TKI following the FDA approval of osimertinib in 2018. These changes in treatment patterns as new treatments became available reinforces the importance of describing contemporary real‐world treatment patterns using the most recently available data. Moreover, the results of this real‐world study demonstrated a longer median 1L treatment duration with osimertinib (17.8 months) as compared with earlier generation EGFR TKIs (8.7 months for first‐generation and 10.5 months for second‐generation EGFR TKI) in the real‐world setting. Other retrospective database studies conducted using US claims data have reported similar median 1L treatment durations for first‐ and second‐generation EGFR TKIs, including 7.7–8.2 months for a combined cohort of EGFR TKIs^13^ and 9.9 months for erlotinib and 12.1 months for afatinib.^12^ Furthermore, time to treatment discontinuation has previously been shown to be a proxy for PFS^23^ and we indeed found that the median durations of first‐ and second‐generation EGFR TKI treatments in this study were comparable to the median PFS reported in their respective clinical trials (9.7–13.3 months^3^, ^24^, ^25^ for erlotinib and 11.0–11.1 months^26^, ^27^, ^28^ for afatinib), as well as the median PFS for first‐generation EGFR TKIs reported in the phase III FLAURA trial (10.2 months).^29^ Lastly, the median duration of 1L osimertinib treatment in our cohort was similar to the median PFS reported in the FLAURA trial and a recent prospective, observational, real‐world study of nine Italian oncology centers (18.9 months in both studies).^9^, ^30^

During the follow‐up period, we observed that patients in the osimertinib cohort had a lower frequency of incident brain or spinal cord metastasis compared with patients treated with other 1L EGFR TKIs (12.8% vs. 16.2%–24.5%), which is consistent with the protective effect of osimertinib against CNS progression reported in the FLAURA trial for patients treated with osimertinib (20%) compared with patients treated with gefitinib or erlotinib (39%)^31^ due to osimertinib's greater penetration of the blood–brain barrier.^32^ However, additional analyses using clinical data would be needed to confirm our findings to account for the longer available follow‐up time for the first‐ and second‐EGFR TKI cohorts and for the timing of the brain or spinal cord metastasis diagnosis during follow‐up since diagnoses occurring close to the index date may suggest baseline metastatic disease that was not coded.

Subsequent 2L therapy was observed in about 30% of patients with first‐ or second‐generation EGFR TKIs in 1L. Among these patients, 2L osimertinib monotherapy was most common (58.3%–60.7%), followed by other EGFR TKIs (28.6%–30.1%), and chemotherapy alone or chemotherapy plus IO therapy (6.0%–7.5%). The frequency of 2L osimertinib is similar to the findings from a recently published retrospective multi‐center study using data from 2014 to 2019 which also reported that osimertinib was the most common 2L treatment option after 1L earlier generation EGFR TKIs, with 65.8% of patients progressing on other EGFR TKI and osimertinib accounting for 55.7% of subsequent treatments.^33^ However, the frequencies of 2L osimertinib and chemotherapy contrast with the treatment patterns observed in previously published data reported from clinical trials and real‐world settings; those studies generally reported lower frequencies of 2L osimertinib and higher frequencies of chemotherapy, which may be a reflection of the study periods ending pre‐2018.^34^, ^35^

The key strengths of this study include the use of linked large pharmacy and medical claims databases which allow for a comprehensive view of treatment patterns. These databases are also widely representative of a variety of insured populations and age categories, so the study findings are anticipated to be generalizable to all mNSCLC patients treated with 1L EGFR TKIs in the US. Lastly, by leveraging the most recently available data at the time of the study start, our findings provide insights into contemporary treatment patterns in real‐world patients, who typically differ from those described in clinical trials due to their stringent eligibility criteria.^36^ Nevertheless, there are several limitations inherent to a retrospective study using claims data, including potential misclassification of diagnosis records, data entry error, and lack of clinical data on disease staging and mutation status. Furthermore, the duration of first‐ and second‐generation EGFR TKIs in this study may be underestimated due to unmeasured confounders not available in the claims databases, such as acquired resistance due to the T790M mutation^37^ and reasons for treatment switching, including intentional switching from earlier generation EGFR TKI to osimertinib before disease progression.^38^ Continuous enrollment cannot be confirmed in the open claims databases, so treatments may be underreported, and treatment durations may be underestimated. However, proxies for continuous enrollment were applied for defining follow‐up time to ensure the patients' pharmacies and medical providers consistently contributed data to the databases. In addition, any treatments received in the inpatient setting were not captured in LRx or Dx. Another important limitation is the imbalance in follow‐up time across the study cohorts. To account for the imbalances in follow‐up in this study, we conducted multiple sensitivity analyses of 1L treatment duration and in these analyses, 1L osimertinib was consistently associated with longer treatment duration compared to other EGFR TKIs, suggesting robustness in our primary analysis results. Due to shorter median follow‐up time for osimertinib compared to other EGFR TKIs, future studies with extended follow‐up are therefore warranted.

## 5 CONCLUSIONS

This study demonstrated that treatment durations are longer with osimertinib compared to other EGFR TKIs used in the 1L setting for the treatment of EGFRm mNSCLC. These findings using real‐world US claims data corroborate clinical trial data from FLAURA and hence reinforce osimertinib's status as the preferred 1L treatment. Future studies with longer follow‐up are still recommended to gain more mature data on 1L osimertinib treatment duration and to better inform 2L treatment patterns following progression on 1L EGFR TKIs.

## AUTHOR CONTRIBUTIONS

Rahul Shenolikar: conceptualization, writing – review and editing. Sizhu Liu: conceptualization, writing – review and editing. Anne Shah: writing – review and editing, project administration. Jenny Tse: conceptualization, writing – original draft, visualization. Yao Cao: methodology, software, formal analysis, writing – review and editing. Aimee Near: conceptualization, writing – original draft, supervision.

## FUNDING INFORMATION

Financial and material support for this study was provided by AstraZeneca, USA.

## CONFLICT OF INTEREST

Rahul Shenolikar and Anne Shah are employed by AstraZeneca and own AstraZeneca stock. Sizhu Liu was employed by AstraZeneca at the time of this study and he is currently employed by BeiGene USA, Inc. Jenny Tse and Aimee Near are employed by IQVIA, which received funding from AstraZeneca to conduct this study. Yao Cao was employed by IQVIA at the time of this study.

## ETHICAL APPROVAL STATEMENT

This was a retrospective claims database study using de‐identified, HIPAA‐compliant data. Ethics approval was not required.

## Supporting information


Table S1

## Data Availability

Research data are not shared.
