# Supplementary material for: Real‐world treatment patterns of metastatic non‐small cell lung cancer patients receiving epidermal growth factor receptor tyrosine kinase inhibitors
Source: Cancer Med. 2022 Jun 15;12(1):159–69. doi: 10.1002/cam4.4918 (PMC9844647; doi:10.1002/cam4.4918)
Supplement: Supplementary file 1 — Table S1 [file CAM4-12-159-s001.docx]

Supplementary table 1: Sensitivity analyses of 1L treatment duration, by index 1L EGFR TKI.

| **Measures** | **Osimertinib**  **N=982** | **First-generation EGFR TKI**  **N=1,060** | **Second-generation EGFR TKI**  **N=463** |
| --- | --- | --- | --- |
| **Main analysis using a gap of >60 days to define treatment discontinuation** | | | |
| Number of patients | | | |
| N (%) | 982 (100.0) | 1,060 (100.0) | 463 (100.0) |
| Time from index to end of follow-up | | | |
| Months, median (Q1, Q3) | 13.5 (7.0, 21.3) | 38.8 (25.2, 51.7) | 35.4 (19.9, 46.8) |
| Reverse-KM follow-up time | | | |
| Months, median (95% CI) | 9.8 (8.9, 10.5) | 20.5 (18.2, 23.7) | 19.3 (15.2, 21.9) |
| Duration of 1L treatment | | | |
| Months, median (95% CI) | 17.8 (15.4, 21.1) | 8.7 (8.0, 9.5) | 10.5 (8.8, 11.5) |
| Number of patients who discontinued 1L EGFR TKI by 12 months**^†^** | | | |
| N (%) | 290 (29.5) | 526 (49.6) | 216 (46.7) |
| Number of patients who discontinued 1L EGFR TKI**^†^** | | | |
| N, (%) | 342 (34.8) | 710 (67.0) | 301 (65.0) |
| Number of patients censored | | | |
| N (%) | 211 (21.5) | 330 (31.1) | 130 (28.1) |
| **Sensitivity analysis using a gap of >90 days to define treatment discontinuation** | | | |
| Duration of 1L treatment | | | |
| Months, median (95% CI) | 24.3 (22.0, NE) | 10.7 (9.4, 11.7) | 11.7 (10.5, 13.9) |
| Number of patients who discontinued 1L EGFR TKI by 12 months**^†^** | | | |
| N (%) | 229 (23.3) | 453 (42.7) | 183 (39.5) |
| Number of patients who discontinued 1L EGFR TKI | | | |
| N (%) | 268 (27.3) | 640 (60.4) | 260 (56.2) |
| **Patients with ≥1 additional fill of the index EGFR TKI and ≥30 days of 1L treatment**^†^ | | | |
| Number of patients | | | |
| N (%) | 857 (87.3) | 874 (82.5) | 390 (84.2) |
| Reverse-KM follow-up time | | | |
| Months, median (95% CI) | 10.4 (9.5, 11.4) | 23.0 (19.5, 29.2) | 20.7 (17.2, 24.9) |
| Duration of 1L treatment | | | |
| Months, median (95% CI) | 20.3 (17.5, 24.3) | 10.3 (9.0, 11.2) | 11.2 (10.2, 12.3) |
| Number of patients who discontinued 1L EGFR TKI | | | |
| N (%) | 271 (31.6) | 599 (68.5) | 258 (66.2) |
| Number of patients censored | | | |
| N (%) | 164 (19.1) | 255 (29.2) | 101 (25.9) |
| **Patients indexed April 2018 or later**^†^ | | | |
| Number of patients | | | |
| N (%) | 863 (87.9) | 73 (6.9) | 105 (22.7) |
| Reverse-KM follow-up time | | | |
| Months, median (95% CI) | 9.0 (8.1, 9.8) | 11.0 (6.2, 16.0) | 10.0 (6.8, 15.2) |
| Duration of 1L treatment | | | |
| Months, median (95% CI) | 17.7 (15.4, NE) | 6.7 (4.0, 8.3) | 9.1 (4.4, 11.6) |
| Number of patients who discontinued 1L EGFR TKI | | | |
| N (%) | 278 (32.2) | 44 (60.3) | 56 (53.3) |
| Number of patients censored | | | |
| N (%) | 176 (20.4) | 18 (24.7) | 23 (21.9) |
| **Patients with ≥12 months of follow-up**^†‡^ | | | |
| Number of patients | | | |
| N (%) | 574 (58.5) | 1,043 (98.4) | 428 (92.4) |
| Reverse-KM follow-up time | | | |
| Months, median (95% CI) | 12.0 (NE, NE) | 12.0 (11.6, 12.0) | 12.0 (11.9, 12.0) |
| Duration of 1L treatment | | | |
| Months, median (95% CI) | NE (NE, NE) | 8.8 (8.0, 9.5) | NE (8.8, NE) |
| Number of patients who discontinued 1L EGFR TKI | | | |
| N (%) | 191 (33.3) | 468 (44.9) | 177 (41.4) |
| Number of patients censored | | | |
| N (%) | 110 (19.2) | 282 (27.0) | 102 (23.8) |
| **Patients with ≥24 months of follow-up**^†‡^ | | | |
| Number of patients | | | |
| N (%) | 190 (19.3) | 1,009 (95.2) | 375 (81.0) |
| Reverse-KM follow-up time | | | |
| Months, median (95% CI) | 24.0 (NE, NE) | 21.5 (18.3, 22.4) | 22.5 (18.1, 24.0) |
| Duration of 1L treatment | | | |
| Months, median (95% CI) | 15.0 (12.5, 20.6) | 8.9 (8.0, 9.7) | 10.9 (8.8, 11.7) |
| Number of patients who discontinued 1L EGFR TKI | | | |
| N (%) | 94 (49.5) | 618 (61.2) | 236 (62.9) |
| Number of patients censored | | | |
| N (%) | 46 (24.2) | 308 (30.5) | 110 (29.3) |

Abbreviations: 1L, First-line; CI, Confidence Interval; Dx, Medical claims database; EGFR TKI, Epidermal growth factor receptor tyrosine kinase inhibitor; LRx, Prescription claims database; Q1, quartile 1; Q3, quartile 3; KM, Kaplan-Meier; NE: not estimable

^†^using a gap of >60 days to define treatment discontinuation.

^‡^Follow-up time is defined as the time from the day after the index date to the earlier of- end of study period, end of stable enrollment, last claims date in Dx, or last claims date in LRx (last day of supply end date for any LRx claim).
